# *Tortula murciana* (Pottiaceae, Bryophyta), a New Species from Mediterranean Mountains

**DOI:** 10.3390/plants14243861

**Published:** 2025-12-18

**Authors:** Rosa M. Ros, Olaf Werner, Jesús Muñoz, Mahmoud Magdy

**Affiliations:** 1 Department of Plant Biology, Faculty of Biology, University of Murcia, Campus of Espinardo, 30100 Murcia, Spain; werner@um.es (O.W.); mahmoud.magdy@um.es (M.M.); 2 Royal Botanical Garden, RJB-CSIC, Plaza de Murillo 2, 28014 Madrid, Spain; jmunoz@rjb.csic.es

**Keywords:** mosses, nrITS, semi-cryptic species, taxonomy, *Tortula subulata* complex

## Abstract

The genus *Tortula* is one of the most diverse and morphologically complex groups within Pottiaceae. This study presents both morphological and phylogenetic evidence for the recognition of a new species, *T. murciana*, within the *T. subulata* complex. The new species is distinguished by a unique combination of traits, including a translucent leaf lamina, upper laminal cells with 3–7 simple, wart-like papillae (verrucae), and middle laminal cells 16–24(35) µm wide, that are much higher near the costa than at the leaf margins. The ventral epidermal cells of the costa at mid-leaf are quadrate to spherical and inflated. The costa is robust, up to 140 µm wide at mid-leaf and papillose on the dorsal side. The apical cell of the apiculus is typically hyaline and often deciduous. The leaf border is usually absent or poorly developed. The basal membrane of the peristome is 0.70–0.90 mm long, with a reticulate pattern where the lumina are delimited by strongly developed muri ornamented with globose clusters of ear-like lobes (auricles). Phylogenetic analysis of the nuclear ITS region places *T. murciana* within the *T. subulata* complex, clearly distinguishing it from *T. mucronifolia* and *T. subulata* var. *graeffii*, with which it shares the closest morphological similarity. Although most specimens can be identified morphologically, some remain difficult to name, making them a semi-cryptic species. The new species is formally diagnosed, described, illustrated, and compared to similar taxa. A key to all species in the complex is also provided.

## 1. Introduction

The genus *Tortula* Hedw. is one of the most diverse and morphologically complex within Pottiaceae [1,2,3,4]. Following a major revision of the genus concept [1,5], it was reinterpreted that sporophyte characters—such as capsule dehiscence and peristome structure—are not fixed diagnostic traits, but rather stages in a continuum of morphological reduction derived from ancestrally spiraled peristomes. Currently, based on this criterion, *Tortula* comprises 105 species [6]. No comprehensive phylogeny of the genus exists on a global scale. However, several molecular studies have shed light on its evolutionary relationships [7,8,9,10]. Nevertheless, some issues regarding the phylogenetic relationships within the genus *Tortula*, as well as with other closely related genera, remain unresolved [1,7]. More recently, a genomic study based on whole plastome sequences from nine *Tortula* species [11], representing two of the most species-rich and widely distributed sections of the genus, section *Tortula* and section *Cuneifoliae* [12], confirmed the close relationships among the analyzed species; their results supported the monophyly of section *Cuneifoliae* but demonstrated the non-monophyly of section *Tortula*. These findings underscore the need to reassess the historical circumscription of the genus and its infrageneric division considering molecular evidence.

*Tortula* species occupy a broad spectrum of ecological niches, reflecting a high degree of adaptability, which has made the genus the most species-rich within the family Pottiaceae across much of the world, resulting in a cosmopolitan distribution [2]. Nevertheless, most species occur in temperate regions of the Northern Hemisphere [13]. In Europe, 36 species are currently known [14], with southern Europe exhibiting the highest species richness, with 30 species in Italy and 26 in continental Spain [15,16]. Across the Mediterranean Region as a whole, 38 species have been recorded [17]. This area is considered a hotspot of diversity [18,19] owing to its distinctive climate, characterized by strongly contrasting seasons with hot, dry summers and mild, wet winters; such conditions exert a marked influence on the bryophyte flora, favoring species with winter-annual life cycles, which emerge during the rainy season and disappear in summer [20]. These climatic features also promote the development of a broad variety of habitats where *Tortula* species thrive, supported by a wide array of desiccation-tolerance traits [21,22,23,24,25].

In addition, *Tortula* species richness is remarkable in high-mountain environments [26,27,28,29,30]. Despite prevailing cold and dry conditions and short growing seasons, these mosses succeed owing to their morphological adaptations, particularly the cushion growth form, which cover the dirt and provide protection from erosion, together with the winter-annual life strategy [31,32].

Within the genus *Tortula*, the so-called *T. subulata* complex [9,33] is a small group of species that in Europe also include *T. inermis*, *T. mucronifolia*, and *T. schimperi*. These species share the presence of relatively large leaves, usually 2.2–5.9 mm long, with no hair point (generally mucronate or apiculate) and a long capsule with twisted peristome with a conspicuous, tessellated basal membrane measuring 0.5–1.6 mm [33,34]. *Tortula subulata* itself is highly variable in morphology, and several varieties have been described, which complicates its distinction from the other species in the complex [33,35,36,37]. However, the morphometric and molecular study by Cano, Werner and Guerra [9] showed that the high morphological and genetic variation within the *T. subulata* complex does not justify recognition of any variety as separate species but rather suggests that these taxa may represent lineages in the midst of a speciation process.

Cryptic species comprise two or more genetically distinct taxa that were historically treated as a single nominal species [38]. This phenomenon represents a hidden yet important component of bryophyte diversity [38,39,40], and it has been frequently detected across all groups and families of bryophytes. The use of molecular methods—particularly DNA barcoding and chloroplast genome analysis—has proven crucial for uncovering hidden diversity and achieving accurate species delimitation [41]. In many cases, once genetic divergence has been detected, a thorough morphological re-evaluation has revealed at least one distinguishing trait between the genetic lineages, e.g., [42,43,44]. This trend has become increasingly common in recent years in Pottiaceae genera, leading to the description of previously overlooked species in genera such as *Aloina* [45], *Oxystegus* [46], *Pseudocrossidium* [47], *Tortella* [48,49,50]. Cryptic speciation has also been detected within the genus *Tortula*. *Tortula muralis* Hedw. exemplifies cryptic diversity, with *rps*4 data revealing distinct haplotypes and regional genetic structure; the presence of unique Japanese lineages and observed paraphyly suggest limited gene flow and the possible existence of hidden species [8,10]. Similarly, Cano, Werner and Guerra [9] noted that *T. mucronifolia* may represent a case of cryptic speciation, where two morphologically indistinguishable taxa are genetically distinct. However, neither of these studies recognized any new species.

While studying members of the Pottiaceae inhabiting Mediterranean mountain areas, we found specimens belonging to the *T. subulata* complex with an unknown morphological combination of traits. A detailed examination of their morphology and ITS sequences revealed that they do not match any known moss species, confirming that they represent an undescribed taxon. Consequently, we here treat them as a new species, which is described, illustrated, and compared with similar and closely related taxa.

## 2. Results

### 2.1. Taxonomy

**Figure 1:**
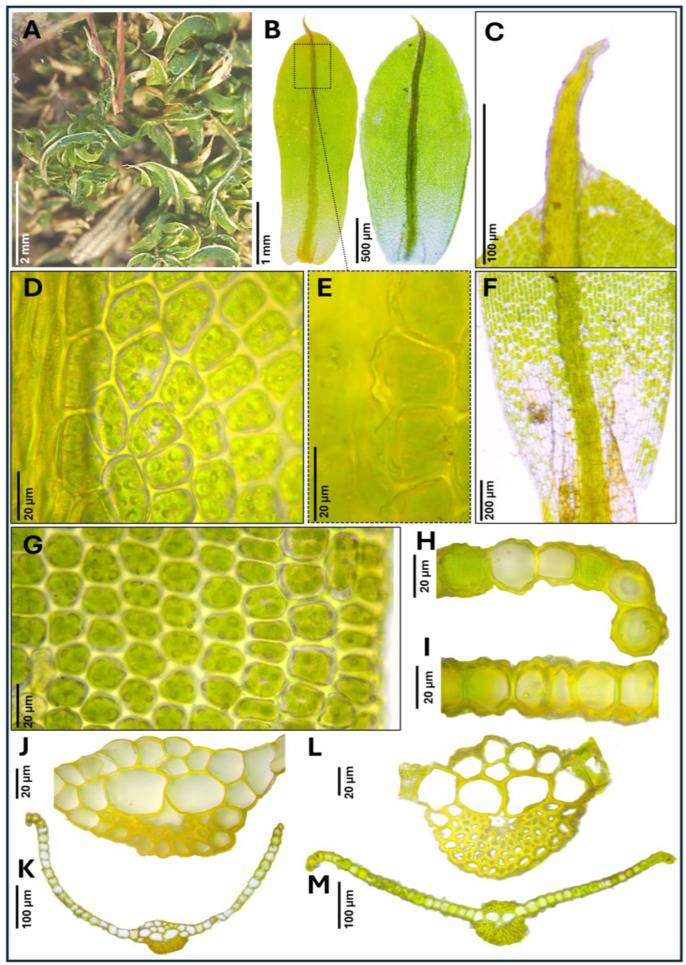
*Tortula murciana* Ros & O. Werner, *sp. nov.* light microscope images of leaves. (**A**) Dry plants showing the leaves individually spirally twisted. (**B**) Complete leaves. (**C**) Leaf apex showing apiculus without the apical cell. (**D**) Upper laminal cells bearing 3–7 simple, wart-like papillae (verrucae). (**E**) Ventral epidermal cells of the costa at the upper part of the leaf showing quadrate, inflated and papillose cells. (**F**) Basal part of the leaf. (**G**) Middle laminal cells bearing 3–7 simple, wart-like papillae (verrucae) and middle marginal cells smooth to scarcely papillose. (**H**) Mid-leaf cross-section detail showing middle marginal cells. (**I**) Mid-leaf cross-section detail showing middle laminal cells bulging. (**J**) Basal costa cross-section biconvex. (**K**) Basal leaf cross-section. (**L**) Costa cross-section at mid-leaf showing ventral epidermal cells inflated and papillose. (**M**) Mid-leaf cross-section showing laminal cells higher near the costa than in the margin. (**A**–**G**) From holotype MUB 63607; (**H**,**J**–**L**) from MUB 63608; (**I**,**M**) from MUB 63617. Photographs by R. M. Ros and M. Magdy.

**Figure 2:**
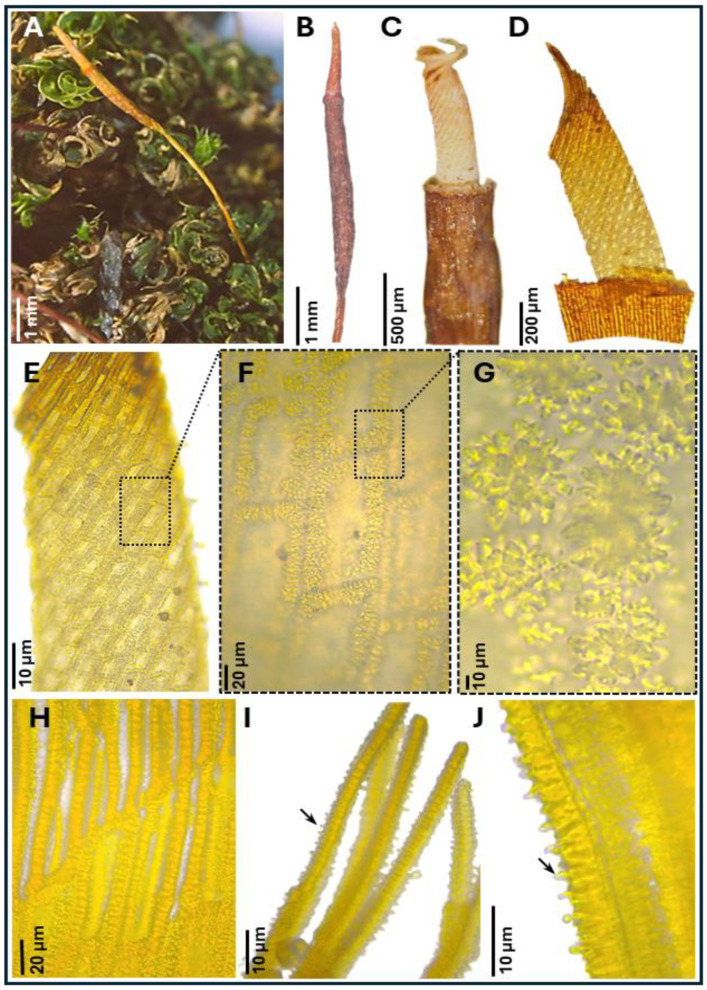
*Tortula murciana* Ros & O. Werner, *sp. nov.* habit and light microscope images of the sporophyte. (**A**) Habit of the plants and sporophyte. (**B**) Capsule showing the cylindrical theca and long conical operculum. (**C**) Upper part of the theca showing the annulus and complete peristome. (**D**) Basal membrane of the peristome with the annulus partially detached. (**E**) Detail of the basal membrane of the peristome showing the tessellated reticulum. (**F**) Detail of the reticulate pattern of the basal membrane of the peristome, with well-developed muri delineating the lumina. (**G**) Ornamentation of the muri of the basal membrane of the peristome, consisting of globose clusters of ear-like lobes (auricles), densely arranged. (**H**) Base of the filiform peristome teeth emerging from the basal membrane. (**I**,**J**) Details of the filiform peristome teeth showing dense papillose ornamentation, with long, non-grouped baculate papillae (arrows). (**A**–**G**) from holotype MUB 63607; (**H**–**J**) from MUB 63609. Photographs by R. M. Ros and M. Magdy.

**Figure 3:**
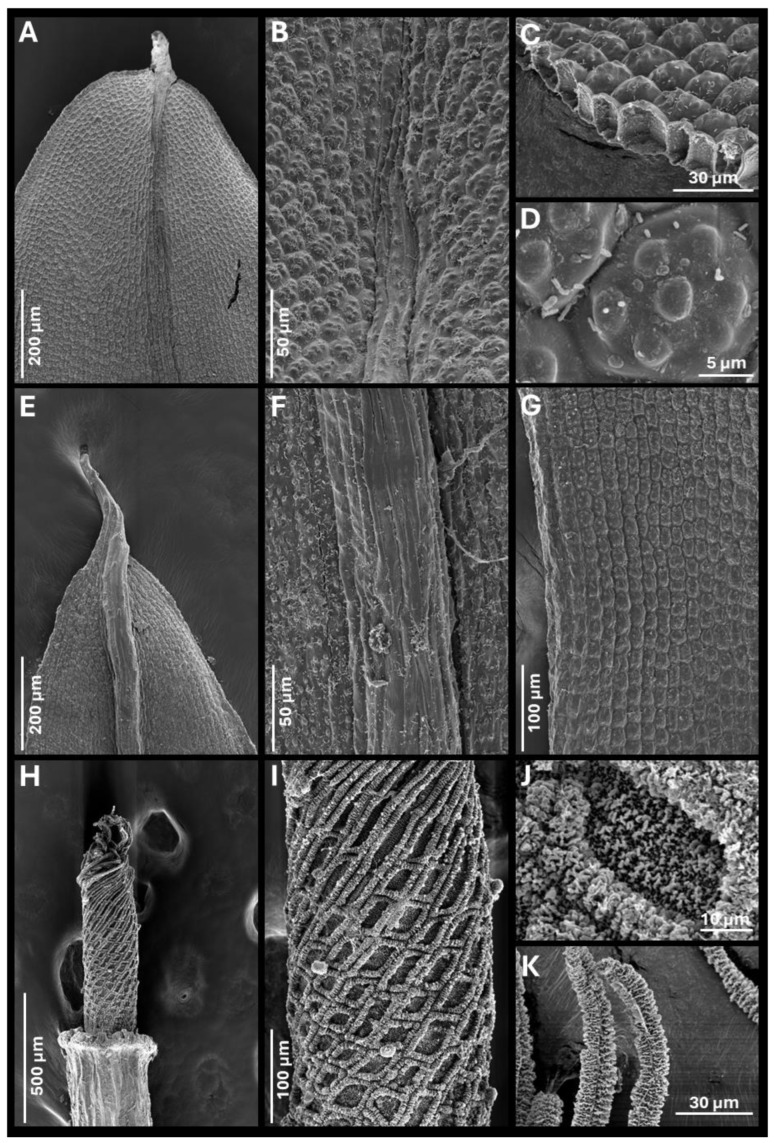
*Tortula murciana* Ros & O. Werner, *sp. nov.* scanning electron microscope images. (**A**) Upper part of the leaf in ventral view. (**B**) Ventral epidermal cells of the costa at the upper part of the leaf. (**C**) Upper laminal cells observed in cross-section. (**D**) Upper laminal cell showing verrucae. (**E**) Upper part of the leaf in dorsal view. (**F**) Dorsal epidermal cells of the costa at mid-leaf. (**G**) Middle part of the leaf in dorsal view showing the leaf margin. (**H**) Peristome. (**I**) Peristome basal membrane showing reticulate pattern. (**J**) Detail of the basal membrane pattern showing the muri and the lumen and ornamentation consisting of globose, densely arranged clusters of ear-like lobes (auricles). (**K**) Peristome tooth detail showing dense papillose ornamentation, with long, non-grouped baculate papillae. All from holotype MUB 63607. Photographs by M. Magdy and R. M. Ros.

***Tortula murciana*** Ros & O. Werner, ***sp. nov.*** (Figure 1, Figure 2 and Figure 3)

- **Diagnosis.** It can be distinguished from other closely related species of *Tortula subulata* complex by the following a unique combination of morphological traits: a translucent leaf lamina, upper laminal cells with 3–7 simple, wart-like papillae (verrucae), 2–3 µm high, middle laminal cells 16–24(35) µm wide, higher near the costa than towards the margins, and ventral epidermal cells of the costa at mid-leaf (when viewed in cross-section) that are quadrate to spherical, inflated, measuring 19.0–24.0 × 14.5–16.0 µm. The costa is robust, up to 140 µm wide at mid-leaf, papillose on the dorsal side, and excurrent in an apiculus, with the apical cell typically hyaline and often deciduous. Leaf border of differentiated cells is usually absent, more rarely poorly developed, non-yellowish, extending up to halfway of the leaf, with 2–3 rows of elongated, smooth cells. Basal membrane of the peristome 0.70–0.90 mm long, with a reticulate pattern where the lumina are delimited by strongly developed muri ornamented with globose, densely arranged clusters of ear-like lobes (auricles).
- **Type.** SPAIN: Murcia province, Revolcadores massif, ascent to Pico de los Obispos, 38.071260 N, 2.268584 W, 1814 m, alkaline, organic-rich soil accumulated between the roots of *Pinus nigra*, 1 August 2023, *R.M. Ros & O. Werner s.n.* (sample ID number 442), (holotype MUB 63607).
- **Description.** *Plants* 0.3–0.5 cm tall, yellowish green, growing in lax turfs; *stems* tomentose, branched; *rhizoids* orange, smooth, without gemmae; *flagelliform shoots* not observed; *stem cross-section* rounded, 350–410 µm wide, hyalodermis absent, scleroderm irregularly present, central strand present, sometimes poorly developed, 40–200 µm wide; *axillary hairs* hyaline, up to eight cells long, basal cell shorter and sometimes brown; *median leaves* usually individually spirally twisted, rarely twisted around the stem when dry, erect when moist, usually elliptical, rarely lanceolate, lingulate or spatulate, frequently concave, 3.3–4.5 × 0.7–1.6 mm, (2.3)2.6–4.5(5.9) times as long as wide; *apex* acute, short acuminate, or obtuse, more rarely rounded, not cucullate; *margins* recurved from base to 2/3 of leaf length, crenulate to papillose-crenulate, unistratose; *leaf border of differentiated cells* usually absent, more rarely poorly developed, non-yellowish, extending up to halfway of the leaf, with 2–3 rows of narrowed, smooth cells; *costa* greenish, gradually narrowing from base to apex, (55)80–140 µm wide at mid-leaf, excurrent in an apiculus (60)200–400(550) µm long, brownish, smooth, with a usually hyaline and deciduous apical cell 50–100 µm long; ventral epidermal cells of the costa at the upper part of the leaf elongated in the 1/4–1/5 upper part of the leaf (laminal cells not covering the costa, exposing ventral stereids), further down quadrate to rectangular, 4–5 cells across the weakly convex ventral surface, inflated and papillose; *dorsal epidermal cells of the costa* at mid-leaf elongate, slightly to strongly papillose; *costa cross-section at mid-leaf* circular to semicircular, with two guide cells in one layer, although sometimes appearing as four guide cells due to each of the two laminal cells on either side of the costa breaks through the costa, band of ventral stereids usually undifferentiated but sometimes present formed by 2 stereid layers, band of dorsal stereids semicircular, with (3)4–5 stereid layers, hydroids differentiated, ventral epidermal cells disposed mostly in one layer, sometimes in two layers, the external usually quadrate to spherical, inflated, 19.0–24.0 × 14.5–16.0 µm, dorsal epidermal cells differentiated; *basal costa cross-section* biconvex; *upper lamina* translucent, collenchymatous, trigones scarcely to strongly developed; *upper laminal cells* usually polygonal, sometimes quadrate, short-rectangular or oblate, (13)16–25(30) × (14)16–28(32) µm, usually thin-walled, sometimes thick-walled, walls 1.2(3.2) µm thick, bearing 3–7 simple, wart-like papillae (verrucae), 2–3 µm high; *upper external marginal cells* quadrate, short-rectangular or oblate, (13)16–24(25.5) × (12)16–24(32) µm [length/width ratio 0.5–2.5], thick-walled, walls 3.2 µm thick; *middle lamina* translucent, collenchymatous, trigones scarcely to strongly developed, rarely absent; *middle laminal cells* usually polygonal or short-rectangular, sometimes quadrate or oblate, (16)20–32(40) × (11)16–24(35) µm, thin-walled, usually equally thin in all cells from costa to margin, occasionally thicker near the costa than in the margin, higher near the costa than towards the margins, bearing 3–9 simple, wart-like papillae (verrucae), bulging in both faces; *middle external marginal cells* short-rectangular, quadrate, oblate or more rarely long-rectangular, (13)16–28(50) × (13)16–23(35) µm [length/width ratio (0.5)0.8–1.8(3.6)], thick-walled, walls 3.2(4.7) µm thick, smooth to scarcely papillose; *basal lamina* not collenchymatous; *basal median laminal cells* rectangular, (35)50–90(110) × (13)20–32(40) µm, thin-walled, that collapse very easily, smooth; *basal external marginal cells* longer and narrower, with 1–2 rows ascending the lamina, (20)40–80(105) × (8)11–16(24) µm [length/width ratio, 2–8.7(14.2)], thin-walled, smooth; *basal yuxtacostal cells* rectangular (50)70–100(150) × (8)16–24(32) µm, thin-walled, and that do not collapse easily. Autoecious. *Perigonia* along the stem. *Perichaetia* apical and subapical. *Perichaetial leaves* undifferentiated or slightly differentiated, more widely elliptical than median leaves or more rarely lingulate, spathulate or ovate, frequently convolute, (2.0)3.0–3.5(4.5) × (0.8)1.0–1.5 mm. *Seta* erect, 8.0–12.4 mm long, straight or sinistrorse below, dextrorse above, yellowish to orange, smooth, in cross-section with epidermal cell walls thickened, cortex cell walls usually homogenously thickened and sometimes thicker on the outside part and gradually thinning to the inside, central strand present, 16.0–25.0 µm in diameter. *Capsule* erect, stegocarpous, exerted; theca cylindrical, 3.0–3.9 × 0.5–0.7 mm, orange; *exothecial cells* rectangular, disposed in rows, frequently with longitudinal walls thicker than transversal, sometimes thin-walled; *annulus* falling only when dissecting, formed by two rows of vesiculose cells, the upper one 16.0–22.0 µm long, reddish externally, and 40.0–47.6 µm, hyaline, when observed in top view; *stomata* phaneroporous, in one or two rows in the neck of the capsule; *peristome basal membrane* tessellated, 0.7–0.9 mm long (measured from the inner side of the capsule), with a reticulate pattern where the lumina are delimited by strongly developed muri ornamented with globose, densely arranged clusters of ear-like lobes (auricles), and 32 *filiform peristome teeth* spirally twisted in two turns, (0.4)0.7–0.9 mm long, orange, densely papillose, with non-grouped, long baculate papillae; *operculum* long conical, (1.3)1.5–2.3 mm long, not systylious, with spirally twisted cells. *Calyptra* cucullate, smooth, 3.5–5.3 mm long, brownish yellow. *Spores* (11)14–20 µm in diameter, finely and irregularly papillose. Leaf color reaction with KOH yellowish.
- **Etymology.** The specific epithet *murciana* refers to the Spanish Region of Murcia, the first and so far only area where the species has been found, located along the Mediterranean Sea. Although it may eventually be detected in other areas, the name highlights the locality where the species was first discovered.
- **Paratypes.** SPAIN: Murcia province, Revolcadores massif, ascent to Pico de los Obispos, 38.071260 N, 2.268584 W, 1814 m, soil accumulated between the roots of *Pinus nigra*, 1 August 2023, *R.M. Ros & O. Werner s.n.* (sample ID number 443), (MUB 63617); *idem*, 38.071937 N, 2.270092 W, 1775 m, alkaline soil accumulated among *Pinus nigra* roots, 15 June 2023, *R.M. Ros & O. Werner s.n.* (sample ID number 365), (MUB 63608); *idem*, 38.072343 N, 2.270303 W, 1749 m, alkaline soil at the base of a *Pinus nigra*, 1 August 2023, *R.M. Ros & O. Werner s.n.* (sample ID number 440), (MUB 63609); *idem*, 38.071978 N, 2.269992 W, 1755 m, alkaline soil accumulated among exposed *Pinus nigra* roots, 1 August 2023, *R.M. Ros & O. Werner s.n.* (sample ID number 446), (MUB 63610); *idem*, 38.072372 N, 2.270413 W, 1743 m, alkaline soil accumulated over exposed *Pinus nigra* roots, 1 August 2023, *R.M. Ros & O. Werner s.n.* (sample ID number 447), (MUB 63611); *idem*, 38.082104 N, 2.24175 W, 1540 m, thin layer of soil over limestone, 15 July 2025, *R.M. Ros, O. Werner &A. Calvo-Torralbo s.n.* (sample ID number 900), (MUB 63612); *idem*, 38.0722770 N, 2.2699746 W, 1759 m, on soil deposited over a limestone bedrock, 16 July 2025, *R.M. Ros, O. Werner &A. Calvo-Torralbo s.n.* (sample ID number 907), (MUB 63613); *idem*, 38.0722770 N, 2.2699746 W, 1759 m, soil deposited over a limestone bedrock, 16 July 2025, *R.M. Ros, O. Werner &A. Calvo-Torralbo s.n.* (sample ID number 908), (MUB 63614); *idem*, 38.071584 N, 2.269359 W, 1782 m, soil in a crevice of limestone rock, 16 July 2025, *R.M. Ros, O. Werner &A. Calvo-Torralbo s.n.* (sample ID number 909), (MUB 63615); *idem*, 38.070131 N, 2.264594 W, 1950 m, soil in a crevice of limestone rock, 16 July 2025, *R.M. Ros, O. Werner &A. Calvo-Torralbo s.n.* (sample ID number 912), (MUB 63616).
- **Distribution and habitat.** *Tortula murciana* is so far restricted to the Revolcadores Massif (Murcia province, SE Spain), where it is relatively abundant on the slopes of Pico de los Obispos, the highest peak in the region (2014 m a.s.l.). The massif has a karstic relief on dolomites and dolomitic limestones, resulting in calcium-rich soils [51]. At higher elevations, the vegetation is dominated by *Pinus nigra* J.F. Arnold, with *Quercus rotundifolia* Lam., *Q. coccifera* L., and a shrub layer of *Berberis hispanica* Boiss. & Reut, *Erinacea anthyllis* Link., and *Genista scorpius* DC. These areas consist of wind-exposed rocky slopes that receive 500–700 mm/m^2^ of rainfall. The species occurs in soil accumulated in rock crevices and among exposed roots of *P. nigra*, microhabitats that protect it from snowmelt runoff and strong insolation. It has been recorded between 1540 and 1950 m a.s.l. In the same habitats *T. inermis*, *T. mucronifolia*, and *T. subulata* also occur.

### 2.2. Phylogenetic Analysis

For this study we obtained 20 new sequences: three of *T. inermis*, four of *T. mucronifolia*, six of *T. subulata s.l.*, and seven of *T. murciana*, which were added to the data published in [9]. The average p-distance of sequence pairs within species is given in Table 1. All sequences of *T. murciana* were identical (p-distance 0.0000) and *T. subulata s.l.* had the highest diversity with an average p-distance of 0.0135 between samples. *Tortula inermis, T. mucronifolia* and *T. schimperi* showed intermediate values of 0.0048, 0.0027 and 0.0124, respectively. The p-distances per sequence pair from averaging over all sequence pairs between species are shown in Table 2. In all cases, the values were higher than the within species values. The lowest value was observed in the comparison of *T. subulata s.l.* and *T. inermis* (0.0144). The mean distance of *T. subulata s.l.* and *T. murciana* was higher with a value of 0.0226 and the mean p-distance between *T. inermis* and *T. murciana* was 0.0191.

**Figure 4:**
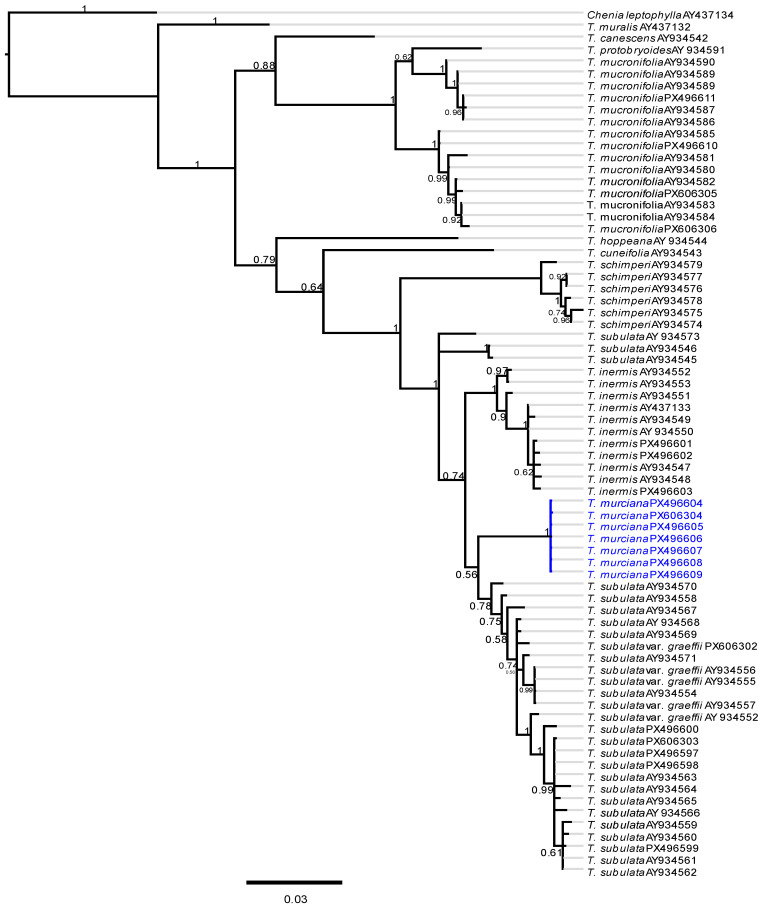
Bayesian phylogenetic tree illustrating the evolutionary relationships within the *Tortula subulata* complex based on sequences from the internal transcribed spacer (ITS) region of ribosomal DNA. The analysis places *T. mucronifolia* near *T. protobryoides,* while *T. schimperi* is resolved as sister to a clade comprising the morphologically distinctive *T. inermis* and *T. murciana*, along with a large group of specimens identified as *T. subulata* or *T. subulata* var. *graeffii.* The relationships within this group could not be resolved in this study. The clades containing samples of *T. inermis* and *T. murciana* exhibit robust statistical support (posterior probability = 1.0), underscoring the reliability of these groupings. The scale bar represents 0.03 substitutions *per* site.

The Bayesian tree (Figure 4) is generally very similar to the tree obtained by [9]. The nrITS sequences separate *T. mucronifolia* clearly from the morphologically similar species of the *T. subulata* complex. Within this complex, *T. schimperi* is sister to the remaining species. *Tortula subulata s.l.* is paraphyletic with *T. inermis* and *T. murciana* nested within the *T. subulata s.l.* assemblage but situated in a clearly distinct clade with high support (posterior probability 1.0).

## 3. Discussion

The *Tortula subulata* complex has puzzled bryologists for over a century. As early as 1912, the first study describing the morphological variability of *T. subulata* and comparing it with *T. mucronifolia* was published [35]. Many later works have echoed this difficulty, often noting the presence of intermediate forms [9,34,37,52,53].

**Figure 5:**
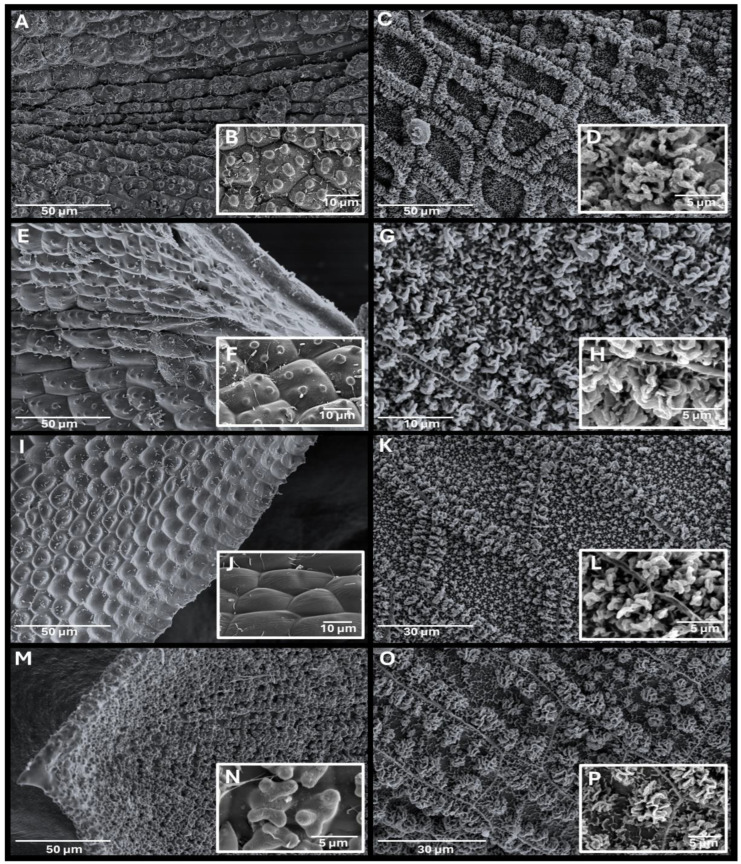
Diagnostic character comparison among *Tortula murciana*, *T. subulata* var. *graeffii*, *T. mucronifolia*, and *T. subulata s.s.*, scanning electron microscope images. *Tortula murciana* (**A**–**D**, holotype MUB 63607), *T. subulata* var. *graeffii* (**E**–**H**, MUB 65489), *T. mucronifolia* (**I**–**L**, TRH B-772918), *T. subulata s.s*. (**M**–**P**, MUB 65516). (**A**,**E**,**I**,**M**) Upper laminal cells in ventral view. (**B**,**F**,**J**,**N**) Upper laminal cell detail. (**C**,**G**,**K**,**O**) Peristome basal membrane reticulate pattern. (**D**,**H**,**L**,**P**) Peristome basal membrane ornamentation detail. Photographs by M. Magdy and R. M. Ros.

The new species, *T. murciana*, clearly belongs to this species complex, as evidenced by its morphological similarity to other members of the group and the nrITS sequence data. Its leaves are sparsely papillose on both surfaces, and translucent in the middle and distal lamina. These characters place the species close to *T. mucronifolia* and some morphs *of T. subulata*, in particular to *T. subulata* var. *graeffii* (Warnst.) Giacom. A comparison of the three taxa is presented in Table 3 and in Figure 5.

The new species can be distinguished from *T. mucronifolia* (Figure S1) primarily on leaf papillosity, having more papillose distal and middle laminal cells, bearing 3–7 simple verrucae (2–3 µm high). In contrast, *T. mucronifolia* has upper laminal cells smooth or more rarely with 1–2 simple conic papillae, about 1 µm high, which are extremely scarce and restricted to a few cells. Additional diagnostic differences are as follows: The upper laminal cells are wider in *T. murciana*, reaching (14)16–28(32) µm wide, whereas in *T. mucronifolia* they are (11)14–20(28) µm wide. The apical cell of the apiculus is somewhat different. In *T. murciana*, it is usually hyaline, very frequently deciduous (most of the dissected leaves for microscopic study lack the apical cell), and 50–100 µm long. In *T. mucronifolia*, it is usually yellowish, only occasionally deciduous, and shorter (30–70 µm long). The ventral epidermal cells of the costa at mid-leaf (seen in cross-section) are quadrate to spherical and inflated in *T. murciana*, measuring 19–24 × 14.5–16 µm. In *T. mucronifolia*, they are quadrate to short-rectangular, elliptical or oblate, not or only slightly inflated, and smaller, measuring 9–13(20) × 8–13(20) µm. The dorsal epidermal cells of the costa at mid-leaf are usually strongly papillose in *T. murciana*, and smooth in *T. mucronifolia*. In cross-section, the middle laminal cells in *T. murciana* are bulging on both surfaces and are approximately twice as tall near the costa (up to 32 µm) as at the margins (up to 16 µm). In *T. mucronifolia*, this difference is less pronounced, or the cells may even be similar in height, reaching up to 22 µm near the costa and 16 µm at the margins. The basal membrane of the peristome in *T. murciana* is 0.70–0.90 mm long, with a distinct reticulation, where the borders form strongly developed muri ornamented with densely arranged, globose clusters of auricles. In contrast, the basal membrane in *T. mucronifolia* is typically shorter, measuring 0.30–0.74(1.0) mm, and the muri are either absent or poorly developed. Additionally, the ornamentation of the muri in *T. mucronifolia* consists of linear clusters of auricles sparsely arranged. Other minor differences between the two species (as shown in Table 3) include the stem tomentum, the length-to-width (L/W) ratio of the median leaves, and the costa width at mid-leaf. In these characters, *T. murciana* has abundantly tomentose stems, a larger L/W ratio (indicating relatively narrower leaves) and a broader costa. Since many of these characters overlap between the two species, confidently assigning some specimens to one or the other can be very challenging.

Warnstorf [35,54] described *T. subulata* var. *graeffii* as a robust form of *T. subulata*, 3 cm tall, densely tufted, with upper leaves broadly lingulate or linear-lingulate, 5–7 mm long and 1–2 mm wide, very finely papillose, yellowish margin, costa strong, projecting into a long mucro, with a darker red seta, often 2–3 cm high, capsule thickened, up to 10 mm long, and yellowish-green spores, 15–18 µm in diameter. European floras have highlighted as diagnostic for *T. subulata* var. *graeffii* the acute or acuminate leaves with poorly developed borders that rarely extend beyond mid-leaf, and the cells weakly papillose and 16–28 μm width at the widest part of the leaf [36,37]. In cultivation, *T. subulata* var. *graeffii* retained its differential characteristics compared to *T. subulata s.s.* [36,55], suggesting a possible genetic basis and even the potential to be treated as a separate species. However, other authors did not find nrITS support for this distinction [9]. Additionally, the latter also reported specimens with intermediate characteristics. Consequently, they considered it to represent mere morphological variation within *T. subulata* and, therefore, lacking taxonomic value. This opinion has been adopted by subsequent authors of floras and checklists, e.g., [14,17,24,56,57], with the notable exception of [25].

*Tortula murciana* shares with *T. subulata* var. *graeffii* (Figure S2) the sparsely distributed papillae on the translucent lamina, but it is generally of smaller stature. Plants of *T. murciana* do not exceed 0.5 cm in height, with leaves 3.3–4.5 mm long and a length-to-width (L/W) ratio of (2.3)2.6–4.5(5.9), whereas var. *graeffii* is a more robust plant, reaching up to 3 cm in height, with leaves 3.9–7.0 mm long and an L/W ratio of 3.9–5.1. They also differ in various leaf traits, including shape, apex, margins, and the leaf border. In *T. murciana*, leaves are elliptical—rarely lingulate, lanceolate, or spathulate—with an apex that is acute, shortly acuminate to obtuse, and more rarely rounded, margins are crenulate to papillose–crenulate toward the apex, the leaf border is usually absent, or more rarely poorly developed, not yellowish, and when present, extends up to halfway along the leaf, composed of 2–3 rows of elongated, smooth cells. In contrast, var. *graeffii* has leaves that are broadly lingulate or linear-lingulate to narrowly lanceolate, with an apex that is acute to long-acuminate. The leaf margins are entire, crenulate, or somewhat irregularly dentate toward the apex. The leaf border is always present, poorly developed, yellowish, and extends up to two-thirds of the leaf length, composed of 3–4 rows of cells that are elongated in the middle and basal parts and quadrate to short-rectangular distally. Other diagnostic traits of *T. murciana*, previously mentioned in comparison with *T. mucronifolia*, can also be used to distinguish it from var. *graeffii*. These include the ventral epidermal cells of the costa at mid-leaf (in cross-section), which in var. *graeffii* are quadrate to elliptical or oblate, not or only slightly inflated, 11.0–19.0 × 11.0–14.0 µm, smaller than in *T. murciana*. In addition, the middle laminal cells in var. *graeffii* (in cross-section) are only slightly taller near the costa (up to 28 µm) compared to the margins (up to 20 µm). The dorsal epidermal cells of the costa at mid-leaf are smooth in var. *graeffii*. The theca is much longer (3.8–10 mm), and the reticules of the basal membrane of the peristome lack muri or have them only slight developed.

As is also the case when comparing *T. murciana* with *T. mucronifolia*, many of the characters mentioned above overlap between the two taxa, often making it very difficult to identify specimens based solely on morphological features. Therefore, the concept of cryptic species can be applied in this case [38]. It has also been frequently reported—as in our case with *Tortula*—that although most specimens can be identified using morphological characters, some collections remain difficult or even impossible to name based on morphology alone, and that is because they have been named by some authors as semi-cryptic [42,43,50,58]. Therefore, it is indispensable to have molecular data that supports morphological identification. Although the morphological differences are relatively minor and partially overlap, their consistency across different populations has proven to be taxonomically significant. The use of multiple genetic markers is generally considered necessary for accurate species delimitation [59]; nevertheless, the ITS region (nrITS1–5.8S–ITS2) has been successfully used alone for species discrimination in numerous cases, e.g., [8,9,60]. These authors argued that even small but consistent differences in DNA sequences—when supported by a distinct set of morphological characters—can serve as strong evidence for species-level differentiation. In the specific case of this study on *T. murciana*, since the DNA sequence data published by Cano, Werner and Guerra [9] were used as the basis for DNA alignment, it was not possible to generate new additional sequences from the specimens used in this work. To complement the ITS data presented here, a more in-depth study is being conducted based on Next-Generation Sequencing methodologies, which will provide additional insights into the genetic diversity and species differentiation in the genus *Tortula* including *T. murciana*.

A challenge encountered during this work was the interpretation of descriptions related to laminal cell papillosity. The description of papillosity in Pottiaceae is often very important. Hedenäs [50] observed that papillae density is an important character for distinguishing *Tortella fragmenta* Hedenäs from *T. fragilis* (Drumm.) Limpr. But terms such as “slightly papillose”, “scarcely papillose”, “inconspicuously papillose” are vague and hinder accurate species differentiation. Even dense papillosity can present in varying degrees. For example, in some cases, papillae are so dense that the boundaries between cells are obscured, while in other cases, despite the cells being strongly papillose, the cell walls remain distinguishable. *Tortula subulata* var. *graeffii* has been described as having inconspicuous papillae, while *T. mucronifolia* can also be, in addition to smooth, inconspicuously papillose. However, the specimens studied of both taxa revealed that although in some cases of both taxa papillae are present, they are different in form and density over the cell wall. The scanning electron microscopy (SEM) analysis of specimens revealed that the papillae are, in fact, different. In var. *graeffii*, the laminal cells are pluripapillose, bearing 2–9 papillae, 1–3 µm high, simple, wart-like sculptural elements wider than tall, which can be referred to as verrucae [61,62]. Due to their width, when observed under a light microscope they may appear as C-shaped papillae; however, since this term is restricted to compound, forked or branched papillae [63,64], it is not of application in this case. In *T. mucronifolia*, the papillae, when present at all, are extremely scarce and restricted to a few cells, with 1–2 simple, small conic protuberances, about 1 µm high, for which the term papillae applies [61,62]. Therefore, the laminal cells of var. *graeffii* should be described as verrucose and those of *T. mucronifolia* as papillose, although their number should also be provided for accuracy. *Tortula murciana* presents the same kind of processes in the laminal cells as var. *graeffii* and should be described as verrucose. The papillosity in *Tortula subulata s.s.* is very different from that previously described. The upper laminal cells are pluripapillose, bearing bifurcate papillae that divide twice, giving the lamina an opaque appearance (Figure S3A–D), and corresponding to which are commonly called C-shaped papillae in bryological literature.

The molecular results presented here are highly congruent with the results presented by Cano et al. [9] for the *Tortula subulata* complex. At a molecular level, *T. mucronifolia* and *T. schimperi* are clearly distinct species. On the contrary, *T. subulata s.l.* appears paraphyletic with the monophyletic and well-supported *T. inermis* and *T. murciana* nested within. But this result is not as surprising as it might seem initially as most scenarios of speciation processes result in paraphyletic taxa in the initial phase when neutral sequences are studied [65]. Based on morphology, van Valen [66] already observed that frequently extant species are direct ancestors of other extant species. Zander [67] defines microgenera that consist of an ancestral species and a few descendant species. A microgenus is a minimally monophyletic group with a paraphyletic direct ancestor of the other species. Zander [67] suggests that about half of the species he studied [68,69] are immediate ancestors of other species. In this sense, *T. subulata s.l.* would be the immediate paraphyletic ancestor of *T. inermis* and *T. murciana*. Additional molecular markers and morphological studies will be needed to show whether *T. subulata* is really paraphyletic or whether the basal samples of this species represent a (semi-)cryptic species.

At this point, *T. murciana* is only known from one mountain range in the Murcia Region of SE Spain. We looked for this species at other Mediterranean Mountain ranges with similar conditions (e.g., Sierra de los Filabres, Sierra de las Nieves) but did not find any specimens at these places. The low genetic diversity detected inside *T. murciana* might indicate a small population size, as under a neutral model, a population’s genetic diversity depends on its effective population size and the gene’s mutation rate [70]. We have found that several *Tortula* species show genetic diversity even within small distances of only several meters (unpublished data). This data might indicate that *T. murciana* is indeed a very rare species. The locality where this species is found is considered a Special Area of Conservation within the Natura 2000 network in the Region of Murcia, and is under no direct threat, but the effect of climate change might put this species in danger as it was only found near the summit of the mountain range.

In the following key, we provide the most useful taxonomic characters for distinguishing *T. murciana* within the *T. subulata* complex (important characters are written in bold).

**1. Upper laminal cells** strongly papillose, **papillae** branched, C-shaped; **leaf lamina** opaque...................................................................................................................................................................**2**
**1**. **Upper laminal cells** smooth or slightly papillose, **papillae** simple, conic or wart-like; **leaf lamina** translucent...........................................................................................................................................................**4**
**2. Leaves** regularly twisted around the stem when dry; **leaf margins** recurved from base to apex; **leaf border** undifferentiated.........................................................................................................................................***T. inermis***
**2. Leave**s generally spirally arranged independently from each other when dry; **leaf margins** plane or recurved from base to 2/3 of the leaf; **leaf border** variable, from distinctly differentiated from base to at least mid-leaf, to almost undifferentiated..................................................................................................................................................**3**
**3. Leaf margins** bistratose; **leaf border** distinctly differentiated from the base to at least mid-leaf; **middle laminal cells** (7.5)10.0–16.3(17.5) µm wide; **peristome basal membrane** (580)1090–1316(1644) µm long....................................................................................................................................................***T. schimperi***
**3. Leaf margins** unistratose; **leaf border** variable, from distinctly differentiated from base to at least mid-leaf, to almost absent; **middle laminal cells** (13.8)15.0–20.0 µm wide; **peristome basal membrane** (400)750–1000(1445) long...........................................................................................................................***T. subulata s.l.***
**4. Upper laminal cells** smooth or with 1–2 simple, conic papillae, 1 µm high, extremely scarce and restricted to a few cells; **leaves L/W ratio** of 1.7–3.6(4.8); **costa width** at mid-leaf 45.5–87.5(114.0) µm; **upper laminal cells** (11)14–20(28) µm wide; **peristome basal membrane** 0.30–0.74(1.0) mm long, with ornamentation consisting of linear clusters of ear-like lobes (auricles), sparsely arranged........................................................................................................................................***T. mucronifolia***
**4. Upper laminal cells** with (0)2–7 simple, wart-like papillae, 1–3 µm high; **leaves L/W ratio** of 3.9–5.1; **costa width** at mid-leaf (55.0)70.0–140.0 µm; **upper laminal cells** 13–28(32) µm wide; **peristome basal membrane** 0.50–0.90 mm long, with ornamentation consisting of globose clusters of ear-like lobes (auricles).............................................................................................................................................................**5**
**5**. **Plants** 1–3 cm tall; **leaves** broadly lingulate or linear-lingulate to narrowly lanceolate, 3.9–7.0 mm long, with acute to long-acuminate apex and entire, crenulate or somewhat irregularly dentate margins towards the apex; **leaf border** always present, poorly developed, yellowish, extending up to 2/3 of the leaf, with 3–4 rows of cells, that are elongated in the middle and basal parts and quadrate to short-rectangular distally; **ventral epidermal cells of the costa at mid-leaf** (seen in cross-section) quadrate to elliptical or oblate, no or very slightly inflated, 11.0–19.0 × 11.0–14.0 µm; **middle laminal cells** (seen in cross-section) slightly higher near the costa (until 28 µm) as in the margin (until 20 µm); **dorsal epidermal cells of the costa at mid-leaf** smooth; **theca** 3.8–10.0 mm long; **basal membrane of the peristome** with a reticulate pattern without or slightly developed muri delimiting the lumina in some areas................................................................................................................***T. subulata (*var. *graeffii*)**
**5. Plants** 0.3–0.5 cm tall; **leaves** elliptical, rarely lanceolate, lingulate or spatulate, 3.3–4.5 mm long, acute or shortly acuminate to obtuse, more rarely rounded apex and crenulate to papillose–crenulate margins at upper part; **leaf border** usually absent, more rarely poorly developed, not yellowish, extending up to halfway of the leaf, with 2–3 rows of elongated, smooth cells; **ventral epidermal cells of the costa at mid-leaf** (seen in cross-section) quadrate to spherical, inflated, 19.0–24.0 × 14.5–1.06 µm; **middle laminal cells** (seen in cross-section) much higher near the costa (until 32 µm) than in the margin (until 16 µm); **dorsal epidermal cells of the costa at mid-leaf** papillose; **theca** 3.0–3.9 mm long; **basal membrane of the peristome** with a reticulate pattern with well-developed muri delimitating the lumina..................................................................................................................................................***T. murciana***

## 4. Materials and Methods

### 4.1. Sampling

Most of the samples on which this study is based were collected as part of bryophyte research conducted by the first two authors (R.M.R. and O.W.), with particular emphasis on the family Pottiaceae. Sampling was carried mainly in the Mediterranean area and more intensively in southern Spain, covering both lowland areas characterized by xeric conditions and xerophytic environments (saline and gypsum soils, and, in general, very sunny and exposed habitats), as well as mountain zones, including rock crevices and proto-soils. Other areas in eastern Spain, Germany, and the Italian Alps were visited with the aim of assessing geographical variability, both at the molecular and morphological levels. Collections were made between 2023 and 2025, mainly during spring and summer (from March to early August), to obtain well-fructified specimens with mature and closed capsules. Based on the authors’ previous experience, some Pottiaceae species may reach this stage between July and August.

Seventy specimens belonging to the *Tortula subulata* complex were selected for careful examination and identification following Smith [36], Cano et al. [9], Cano [34], and Zander & Eckel [24]. These included several specimens of *T. mucronifolia* borrowed from the Norwegian University of Science and Technology herbarium (TRH), the type specimen of *T. mucronifolia* housed at the G herbarium, and the type specimen of *T. subulata* var. *graeffii* from the JE herbarium (Table S1).

A careful morphological study was carried out on the specimens sequenced and not sequenced to assess the differences comparatively between *T. murciana*, *T. mucronifolia*, and *T. subulata* var. *graeffii*. Samples are deposited at MUB herbarium.

Morphological observations were performed with a DM 1000 LED light microscope (LM; Leica, Wetzlar, Germany), as well as with a A8APO stereomicroscope (Leica, Wetzlar, Germany) connected to a DFC295 digital camera (Leica, Wetzlar, Germany), which enabled image capture and transfer to a computer. Image processing and measurements were carried out using the Leica Application Suite software, version 4.1.0. A subset of specimens was further examined through scanning electron microscopy (SEM) to verify leaf papillosity and details of the peristome. For SEM preparation, samples were fixed in McDowell’s fixative for 24 h at 4 °C, washed in 0.1 M cacodylate buffer with 8% sucrose, and then dehydrated in increasing concentrations of acetone before critical point drying. They were mounted on aluminum stubs and sputter-coated with a 5.0 nm platinum layer (EM ACE 600, Leica, Wetzlar, Germany). Analyses were performed using an FE-SEM (Apreo S Lovac IML, Thermo Fisher, Waltham, MA, USA) at 5 kV with secondary electrons. Leaves selected for analysis were consistently taken from the upper and middle portion of the stem.

### 4.2. DNA Sequencing

DNA from one gametophyte was extracted by addition of 100 µL of the dilution buffer supplied with the Phire Plant Direct PCR Kit (Thermo Fisher, Madrid, Spain) and using a Mixer Mill MM400 (Retsch, Haan, Germany) at 30 rpm for 4 min in the presence of two tungsten beads with two mm diameter. The beads were removed and the tubes centrifuged for 1 min at 10,000× *g* to remove cellular debris. The supernatant was stored at −20 °C until further use. The nrITS region was amplified with the primers ITS1 and ITS4 [71] at a final concentration of 0.5 µM. 1 µL of stock DNA was used as a template in 25 µL reaction volume with the Phire Plant Direct PCR Master Mix (Thermo Fisher, Madrid, Spain). The addition of 0.5 µL of 10% skimmed milk powder in water to the reaction led to more robust amplifications [71,72]. The amplification conditions were as follows: 2 min at 98 °C, 35 cycles with 5 s at 98 °C, 15 s at 60 °C and 60 s at 72 °C, and a final 5 min extension step at 72 °C. Amplification products were controlled on 1% agarose gels and successful reactions were precipitated by adding 2.5 µL of 3M Na-acetate and 100 µL of 96% ethanol. After 10 min centrifugation at 10,000× *g* the precipitated DNA was first washed with 70% ethanol and then with 96% ethanol. The dried pellets were dissolved in 20 µL of 10 mM Tris, 0.1 mM EDTA at pH 8.5, and sequenced using the amplification primers.

### 4.3. Data Analysis

The DNA sequence data published by Cano, Werner and Guerra [9] were used as bases for the DNA alignment (GenBank accession numbers AY934542-AY934591) and the 20 newly generated sequences (Table S1) were added and edited using Bioedit v5.0.9 [73] and aligned manually. Between taxa mean distances and within taxa mean distances were calculated with MEGA X [74]. We used the program seqstate [75] to recodify the indels in the data matrix as 0/1 character states according to Simmons and Ochoterena [76]. The data were analyzed by Bayesian inference implemented with MrBayes v3.2 [77,78,79]. Gaps were treated as standard data. Trees were sampled across the substitution model space in Bayesian MCMC analysis itself [80] using the option nst = mixed. Therefore, *a priori* model testing was not necessary. Indels were treated as separate unlinked partitions, using the restriction site model (F81). Two runs with four chains were conducted with 2,000,000 generations. Trees were sampled every 1000th generation and the first 200,000 generations were discarded (burn-in) to exclude the trees before the chain reached the stationary phase. We checked for stationarity of the log likelihood values, that the Potential Scale Reduction Factor (PSRF) was close to “1” (0.99 < PSRF < 1.01), and that the estimated sample size was above 1500. The final trees were edited with TreeGraph v2 [81].

## Figures and Tables

**Table 1:** Estimates of average evolutionary divergence over sequence pairs within *Tortula subulata* complex species. The number of base differences per site from averaging over all sequence pairs within each species are shown. The rate variation among sites was modeled with a gamma distribution (shape parameter = 1). This analysis involved 69 nucleotide sequences.

*T. inermis*	*T. mucronifolia*	*T. murciana*	*T. schimperi*	*T. subulata s.l.*
0.0048	0.0028	0.0000	0.0124	0.0135

**Table 2:** Estimates of evolutionary divergence over sequence pairs between species. The number of base differences per site from averaging over all sequence pairs between species are shown. This analysis involved 69 nucleotide sequences. The distance values of *Tortula murciana* to the remaining species are higher than those of the generally recognized species *T. inermis*. The distances between species are higher than the maximum within species value (0.0135).

	*T. inermis*	*T. mucronifolia*	*T. murciana*	*T. schimperi*	*T. subulata s.l.*
***T. inermis***	-	-	-	-	-
***T. mucronifolia***	0.0501	-	-	-	-
***T. murciana***	0.0191	0.0530	-	-	-
***T. schimperi***	0.0375	0.0468	0.0402	-	-
***T. subulata s.l.***	0.0144	0.0486	0.0226	0.0353	--

**Table 3:** Differential characters for *Tortula murciana*, *T. mucronifolia* and *T. subulata* var. *graeffii.* Characters of *T. mucronifolia* are based on the study of the images of the lectotype, own observations, and several literature sources [9,24,37]. Those of *T. subulata* var. *graeffii* are based on the study of the lectotype, own observations, as well as Warnstorf [35,54], Nyholm [37] and Smith [36]. Diagnostic characteristics for each taxon are written in bold.

Character	*T. murciana*	*T. mucronifolia*	*T. subulata* var. *graeffii*
Plants size (stem)	**0.3**–**0.5 cm**	0.5–1.6 cm	**1.0**–**3.0 cm**
Stem tomentum	abundant	absent or sparse	sparse to abundant
Leaves shape	usually elliptical, rarely lanceolate, lingulate or spathulate	usually elliptical, lingulate or ovate, rarely lanceolate or spathulate	**broadly lingulate, linear-lingulate or narrowly lanceolate**
Median leaves size (L × W)	3.3–4.5 × 0.7–1.6 mm	(1.7)2.5–4.0(5.9) × (0.6)0.7–1.4 mm	**3.9–7.0** × 0.9–2.0 mm
Median leaves L/W ratio	(2.3)2.6–4.5(5.9)	**1.7** **–** **3.6(4.8)**	3.9–5.1
Median leaves apex	acute or shortly acuminate to obtuse, more rarely rounded	acute or obtuse to rounded	**acute to long-acuminate**
Median leaf margins at upper part	crenulate to papillose–crenulate	entire to crenulate	**entire, crenulate or somewhat irregularly dentate towards the apex**
Median leaves border	usually absent, more rarely poorly developed, non-yellowish, extending up to halfway of the leaf, with 2–3 rows of elongated, smooth cells	usually absent, more rarely poorly developed, yellowish, extending up to halfway of the leaf, with 1–3 rows of elongated, smooth or more rarely, scarcely papillose cells	**always present, poorly developed, usually yellowish, extending up to 2/3 of the leaf, with 3**–**4 rows of cells, that are elongated in the middle and basal parts and quadrate to short-rectangular distally**
Apiculus apical cell	**usually hyaline, very frequently deciduous**	usually yellowish, sometimes deciduous	usually greenish, sometimes deciduous
Apiculus apical cell length	50–100 µm	**30–70** **µm**	40–90 µm
Costa width at mid-leaf	**(55.0)80.0**–**140.0 µm**	45.5–87.5(114.0) µm	70.0–100.0 µm
Dorsal epidermal cells of the costa at mid-leaf	slightly to strongly **papillose**	smooth	smooth
Ventral epidermal cells of the costa at the upper part of the leaf (below the most apical elongated cells)	quadrate to rectangular, inflated and papillose	**quadrate to rectangular, not or slightly inflated and smooth**	rectangular, inflated and papillose
Ventral epidermal cells of the costa at mid-leaf (seen in cross-section)	**quadrate to spherical, inflated, 19.0–24.0 × 14.5–16.0 µm**	quadrate to short-rectangular, elliptical or oblate, non or very slightly inflated, 9.0–13.0(20.0) × 8.0–13.0(20.0) µm	quadrate to elliptical or oblate, not or very slightly inflated, 11.0–19.0 × 11.0–14.0 µm
Upper laminal cells width	(14)16–28(32) µm	(**11)14–20(28) µm**	13–27 µm
Upper laminal cells papillosity	3–7 simple, wart-like papillae (verrucae), 2 3 µm high	**absent or, if present at all, extremely scarce and restricted to a few cells, with 1–2 simple, conic papillae, 1 ** **µ** **m high**	2–8 conic, wart-like papillae (verrucae), 1–3 µm high
Middle laminal cells width	**(11)16–24(35) µm**	(10)14–28 µm	16–27 µm
Middle laminal cells papillosity	3–9 simple, wart-like papillae (verrucae)	**smooth (very rarely with 1–2 simple conic papillae)**	4–9 simple, wart-like papillae (verrucae)
Middle laminal cells height (seen in cross-section)	**higher near the costa (until 32 ** **µm** **) than in the margin (until 16 ** **µm)**	similar height or slightly different near the costa (until 22 µm) as in the margin (until 16 µm)	slightly higher near the costa (up to 28 µm) than in the margin (up to 20 µm)
Theca length	3.0–3.9 mm	2.0–3.6(6.0) mm	**3.8–10.0 mm**
Peristome basal membrane length	0.70–0.90 mm	**0.30–0.74(1.0) mm**	0.50–0.85 mm
Pattern of the tessellated peristome basal membrane	**reticulate with strongly developed muri delimiting the lumina**	reticulate without or slightly developed muri delimiting the lumina	reticulate, without or slightly developed muri delimiting the lumina in some areas
Ornamentation of the muri	**globose clusters of ear-like lobes (auricles), densely arranged**	linear clusters of ear-like lobes (auricula), sparsely arranged	globose clusters of ear-like lobes (auricles), sparsely arranged

## Data Availability

The original contributions presented in this study are included in the article/Supplementary Material. Further inquiries can be directed to the corresponding author. The new sequences generated for this work are available at GenBank: PX496597-PX496611, PX606302-PX606306.

## Supplementary Materials

The following supporting information can be downloaded at https://www.mdpi.com/article/10.3390/plants14243861/s1. Figure S1: *Tortula mucronifolia* scanning electron microscope images. Figure S2: *Tortula subulata* var. *graeffii* scanning electron microscope images. Figure S3. *Tortula subulata s.s*. scanning electron microscope images. Table S1: Samples of the *Tortula subulata* complex used in the study with voucher information.
