# Supplementary material for: Tortula murciana (Pottiaceae, Bryophyta), a New Species from Mediterranean Mountains
Source: Plants (Basel). 2025 Dec 18;14(24):3861. doi: 10.3390/plants14243861 (PMC12736775; doi:10.3390/plants14243861)
Supplement: Supplementary file 1 [file plants-14-03861-s001.zip › Suplementary materials_proofs.pdf]

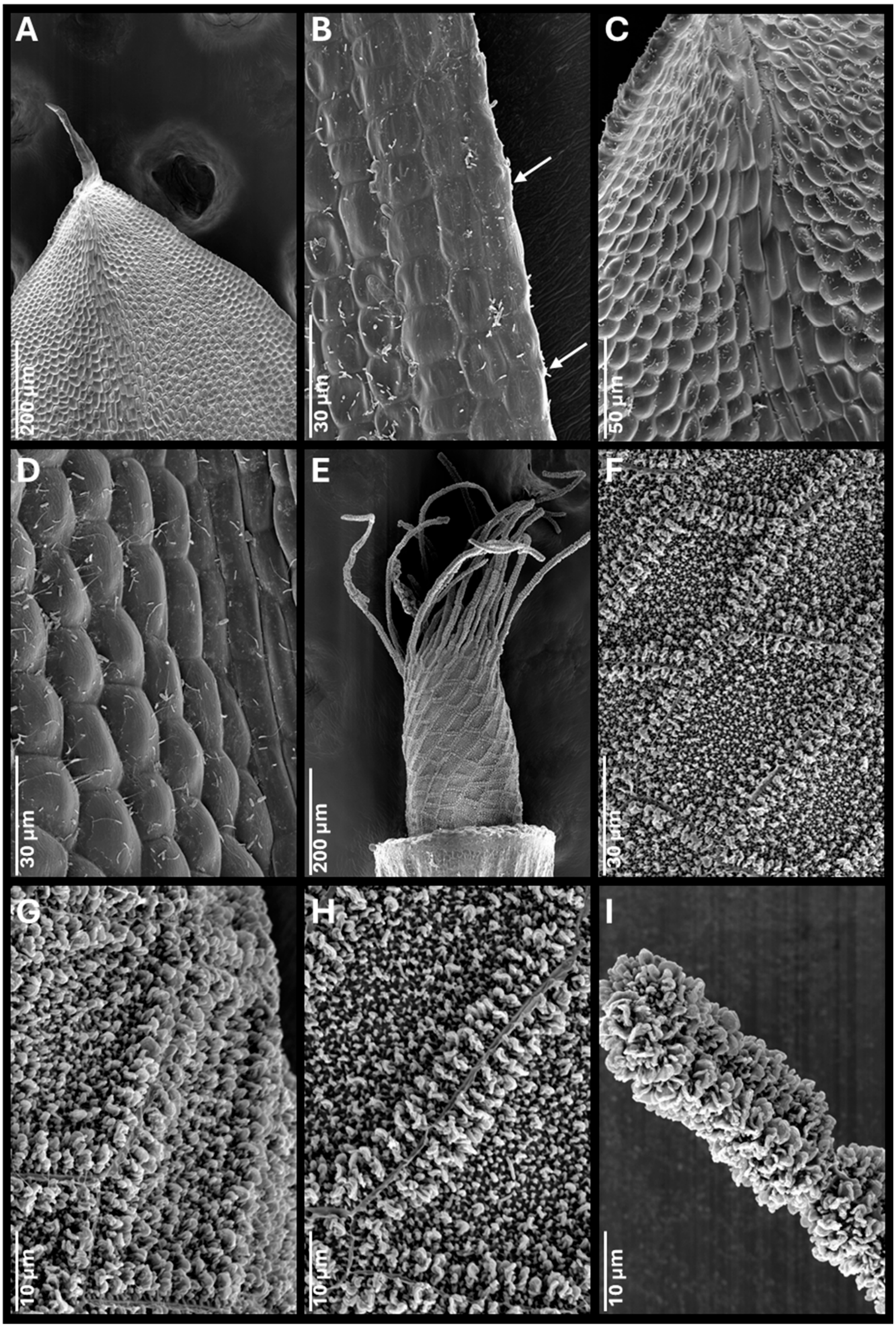

Figure S1. *Tortula mucronifolia* scanning electron microscope images. (A) Upper part of the leaf in ventral view. (B) Leaf margin at the middle part of the leaf, showing some sparse and scattered papillae (arrows). (C) Ventral surface cells of the costa at the upper part of the leaf. (D) Upper laminal cells smooth. (E) Peristome. (F, G) Peristome basal membrane pattern showing reticule with slightly developed muri delimitating the lumina. (H) Peristome basal membrane ornamentation consisting of linear clusters of ear-like lobes (auricles), sparsely arranged. (I) Peristome tooth detail showing ornamentation. All from TRH B-772918. Photographs by M. Magdy and R. M. Ros.

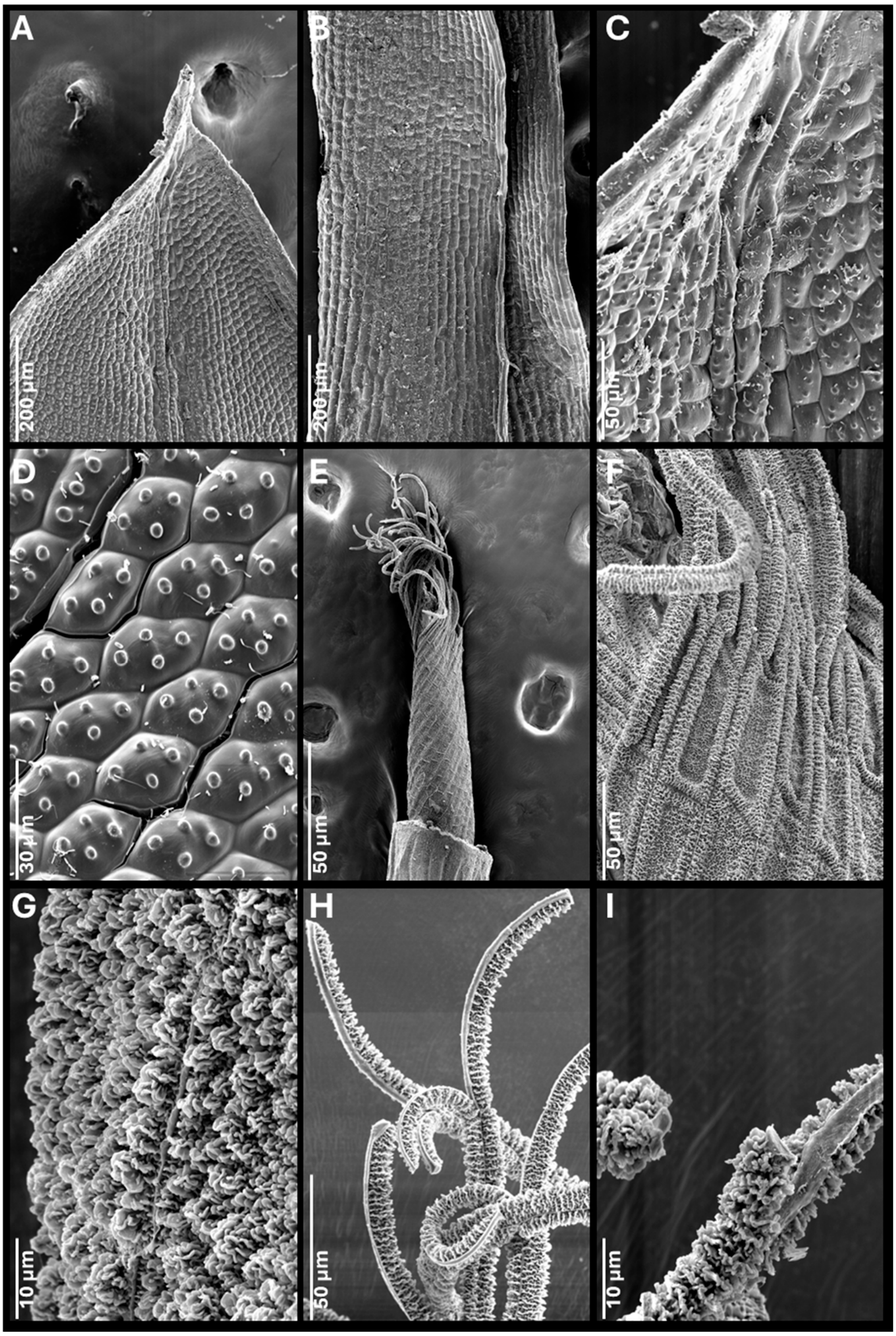

Figure S2. *Tortula subulata* var. *graeffii* scanning electron microscope images. (A) Upper part of the leaf in ventral view. (B) Middle part of leaf in dorsal view showing the leaf margins. (C) Ventral surface cells of the costa at the upper part of the leaf. (D) Upper laminal cell showing papillosity with verrucae. (E) Peristome. (F) Peristome basal membrane showing reticulate pattern with slightly developed muri delimiting the lumina. (G) Detail of the peristome basal membrane showing the reticulum without muri delimiting the lumina, and ornamentation consisting of sparsely arranged, globose clusters of ear-like lobes (auricles). (H) Peristome teeth. (I) Peristome tooth detail showing ornamentation. All from MUB 65489. Photographs by M. Magdy and R. M. Ros.

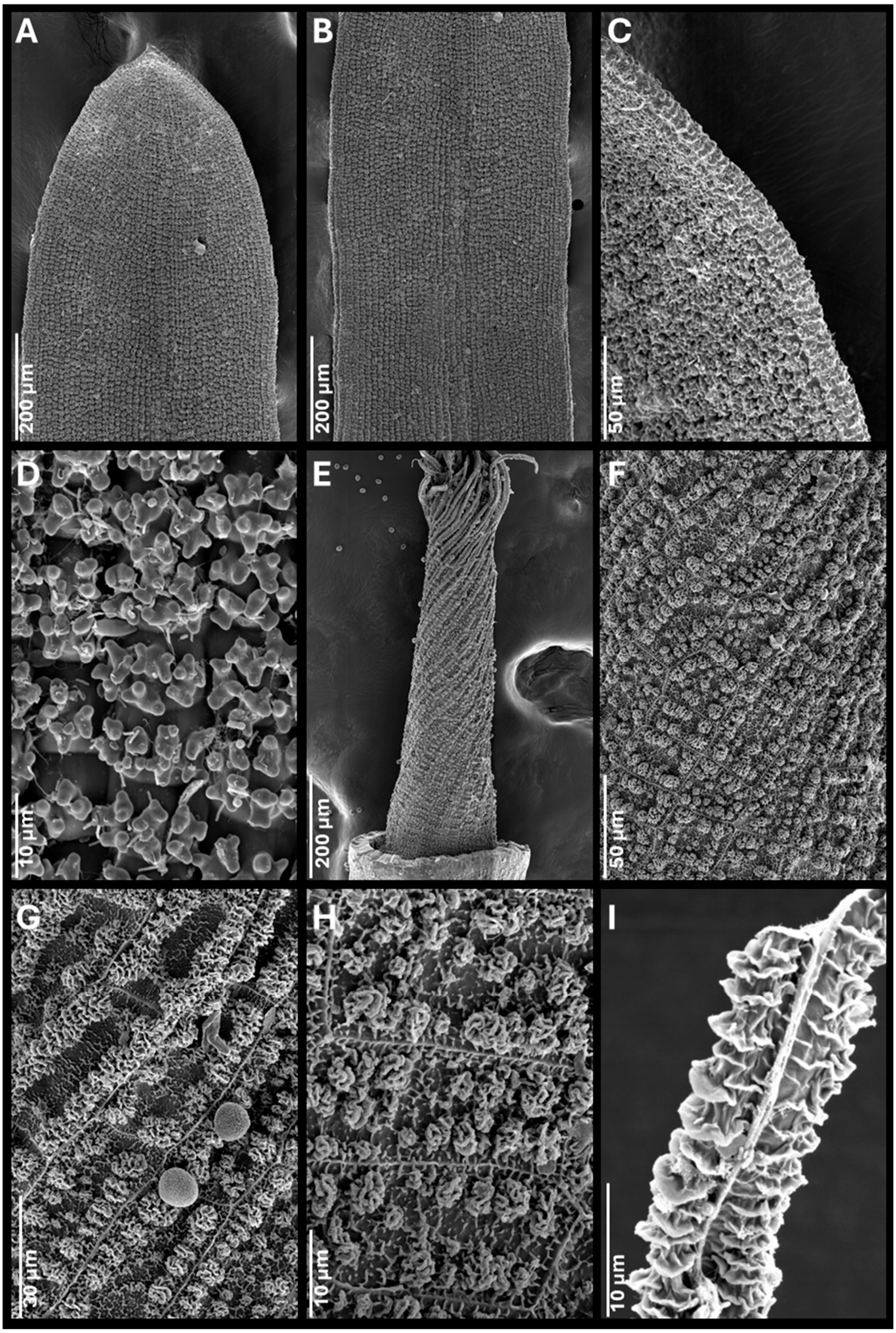

Figure S3. *Tortula subulata s.s.* scanning electron microscope images. (A) Upper part of the leaf in ventral view. (B) Middle part of the leaf in ventral view showing the leaf margins. (C) Leaf margin at upper part of the leaf. (D) Upper laminal cells pluripapillose, bearing bifurcate papillae. (E) Peristome. (F, G) Peristome basal membrane reticulate pattern without or only slightly developed muri delimiting the lumina in some areas. (H) Peristome basal membrane ornamentation consisting of globose clusters of ear-like lobes (auricles), sparsely arranged (I) Peristome tooth detail showing ornamentation. All from MUB 65516. Photographs by M. Magdy and R. M. Ros.

Table S1. Samples of the *Tortula subulata* complex used in the study with voucher information. All samples were examined morphologically. Those whose ITS sequences were also used in the phylogenetic analysis are identified by their GenBank accession number. Literature references are provided for previously published sequences, and the new ITS sequences generated in this study are shown shaded in gray. Samples exhibiting intermediate characteristics are marked.

GenBank accession numbers	Voucher information	Intermediate characteristics
	Tortula inermis	
PX496601	SPAIN: Murcia province, Revolcadores massif, ascent to Pico de los Obispos, 38.071431 N, 2.268970 W, 1797 m, 1 August 2023, *R.M. R.M. Ros & O. Werner s.n.*, sample ID number 441, MUB 65514	
PX496602	SPAIN: Murcia province, Revolcadores massif, ascent to Pico de los Obispos, 38.073466 N, 2.272301 W, 1680 m, 1 August 2023, *R.M. R.M. Ros & O. Werner s.n.*, sample ID number 448, MUB 65515	
PX496603	SPAIN: Murcia province, Revolcadores massif, base of Pico Revolcadores, 38.080669 N, 2.278166 W, 1512 m, 8 April 2023, *R.M. R.M. Ros & O. Werner s.n.*, sample ID number 174, MUB 65513	
	Tortula mucronifolia	
PX496610	NORWAY: Finnmark, Vardø, Vesterelva, Bukkemoltangen, $UTM_{EUREF89}$: 415634,7815379, 78 m, 14 August 2016, *Torbjørn Høitomt 3566Høi, Kristian Hassel*, THR B-89967	
PX496611	NORWAY: Oppland, Sør-Fron, Stebergsberget, $LatLong_{WGS84}$: 61,549673°N 10,006395°E, 15 April 2014, *Anders Breili M3340*, TRH B-89468	
PX606305	SPAIN: Guadalajara province, Alto Tajo Natural Park, road GU-982 from Checa to Barranco la Hoz, past the junction to Cueva del Tornero, 40.552358N, 1.810021 W, 1565 m, 27 Juni 2023, *R.M. Ros & O. Werner s.n.*, sample ID number 371, MUB 65507	
PX606306	ITALY: Trentino-Alto-Adige/Südtirol, Schnalstal/Val Senales, Katharinaberg, 46.689642 N, 10.935743 E, 1258 m, 29 July 2025, *R.M. Ros & O. Werner s.n.*, sample ID number 920, MUB 65485	
AY934584	Cano et al. [9] MUB 14041	
AY934581	Cano et al. [9] MUB 17402	√
AY934582	Cano et al. [9] MUB 13630	√
	Tortula mucronifolia 72, Herbarium Hedwig-Schwägrichen in G, Lectotype, G00042902	
	GREENLAND: East Greenland, Zackenberg, Wollastone Forland, UTM_{WGS84}: WC 12539,63585, 6 m, 30 August 2009, *Kristian Hassel 421, Tommy Prestø*, THR B-692849	
	GERMANY: Baden-Württemberg, Schwäbish Alb, Wiesensteig, junto a Freibad y Wasserwerk, 48.562274 N, 9.609201 N, 610 m, 12 August 2024,, *R.M. Ros & O. Werner s.n.*, sample ID number 535, MUB 65517	√
	ITALY: Trentino-Alto-Adige/Südtirol, Schnalstal/Val Senales, Katharinaberg, 46.689642 N, 10.935743 E, 1258 m, 29 July 2025, *R.M. Ros & O. Werner s.n.*, sample ID number 922, MUB 65487	
	ITALY: Trentino-Alto-Adige/Südtirol, Malles Venosta/Mals, Slingia/Schlinig, 3, paved forest road starting from the village, used for c*R.M. Ros*s-country skiing, 46.707371 N, 10.466140 E, 1790 m, *R.M. Ros & O. Werner s.n.*, sample ID number 942, MUB 65493	

	idem, sample ID number 943, MUB 65494	
	idem, sample ID number 944, MUB 65495	
	ITALY: Trentino-Alto-Adige, Südtirol, Malles Venosta/Mals, Slingia/Schlinig, 4, paved forest road number 1, starting from the village, 46.709948 N, 10.463101 E, 1844 m, *R.M. Ros & O. Werner s.n.*, sample ID number 946, MUB 65496	
	ITALY: Trentino-Alto Adige, Südtirol, Castelrotto/Kastelruth village, Hotel Belbedere-Schönblick wall, 46.565134 N, 11.556871 E, 3 August 2025, *R.M. Ros & O. Werner s.n.*, sample ID number 956, MUB 65493	
	NORWAY: Oppland, Sør-Fron, Skarsberget v Solbrå, UTM: 32V 544144,6828593, 383 m, 30 September 2013, *Tom Hellik Hofton THH 13329*, TRH B-3402	
	NORWAY: Finnmark, Nesseby, Hammernesfjellet S, $UTM_{EUREF89}$: 572518,7783368, 5 m, 1 July 2016, *Torbjørn Høitomt 3937Høi*, TRH B-12600	
	NORWAY: Buskerud, Hole, Frognøya Ø, UTM: 32V 566940,6657600, 73 m, 4 Juni 2014, *Torbjørn Høitomt M1318Høi, Sigve Reiso*, TRH B-37014	
	NORWAY: Buskerud, Hole, Lille Svartøya Ø, UTM:32V 568270,6658400, 69 m, 4 Juni 2013, *Torbjørn Høitomt M1309Høi, Sigve Reisok*, TRH B-37022	
	NORWAY: Oppland, Sør-Fron, Stebergsberget, $LatLong_{WGS84}$: 61,549673°N 10,006395°E, 15 April 2014, *Anders Breili M3565*, TRH B-89615	
	NORWAY: Oppland, Sør-Fron, Stebergsberget, $LatLong_{WGS84}$: 61,549673°N 10,006395°E, 15 April 2014, *Anders Breili M3566*, THR B-89616	
	NORWAY: Finnmark, Alta, Altadalen, mellom Sautso-gården og Torghatten, $UTM_{EUREF89}$: 607210,7740143, 322 m, 6 August 2016, *Torbjørn Høitomt 3499HøiB, Terje Blindheim*, THR B-89908	
	NORWAY: Finnmark, Vardø, Vesterelva, $UTM_{EUREF89}$: 413824,7814357, 101 m, 14 August 2016, *Torbjørn Høitomt 3584Høi, Kristian Hassel*, THR B-89982	
	NORWAY: Sør-Trøndelag, Oppdal, Kongsvoll, mellom Driva og jernbanen rett sør for fjellstua, UTM: 32V 531178,6907890, 849 m, 9 September 2013, *Torbjørn Høitomt M2098Høi, Kristin Wangen, Niklas Lönnell*, THR B-91349	
	NORWAY: Sør-Trøndelag, Oppdal, Driva, Losløkkja, UTM: 32V 0532994,6933120, 696 m, 27 September 2015, *Torbjørn Høitomt 3307 Høi*, THR B-92871	
	NORWAY: Oppland, Vestre Slidre, Lomen - rett vest for Lomen kyrkje, UTM_{WGS84}: 493788,6777516, 143 m, 23 August 2018, *Torbjørn Høitomt 4424Høi*, THR B-108477	
	NORWAY: Hedmark, Ringsaker, Helgeberget, UTM:32V 590442,6758360, 123 m, 5 Juni 2012, *Torbjørn Høitomt M187Høi, Sigve Reiso*, THR B-772918	
	SPAIN: Guadalajara province, road GU-982, c*R.M. Ros*sing the Tajo river, Las Tres Cruces, 40.397996 N, 1.805469 W, 1507 m, *R.M. R.M. Ros & O. Werner s.n.*, sample ID number 397, MUB 65518	√
	SPAIN: Murcia province, subida al Pico del Obispo, 38.071260 N, 2.268584 W, 1814 m, 1 August 2023, *R.M. R.M. Ros & O. Werner s.n.*, sample ID number 444, MUB 63601	√
	Tortula murciana	

PX496604	SPAIN: Murcia province, Revolcadores massif, ascent to Pico de los Obispos, 38.071937 N, 2.270092 W, 1775 m, 15 Juni 2023, *R.M. R.M. Ros & O. Werner s.n.*, sample ID number 365, paratype, MUB 63608	
PX496605	*idem*, 38.071260 N, 2.268584 W, 1814 m, 1 August 2023, *R.M. R.M. Ros & O. Werner s.n.*, sample ID number 442, holotype, MUB 63607	
PX496606	*idem*, 38.071260 N, 2.268584 W, 1814 m, 1 August 2023, *R.M. R.M. Ros & O. Werner s.n.*, sample ID number 443, paratype, MUB 63617	
PX496607	*idem*, 38.072343 N, 2.270303 W, 1749 m, 1 August 2023, *R.M. R.M. Ros & O. Werner s.n.*, sample ID number 440, paratype, MUB 63609	
PX496608	*idem*, 38.071978 N, 2.269992 W, 1755 m, 1 August 2023, *R.M. R.M. Ros & O. Werner s.n.*, sample ID number 446, paratype, MUB 63610	
PX496609	*idem*, 38.072372 N, 2.270413 W, 1743 m, 1 August 2023, *R.M. R.M. Ros & O. Werner s.n.*, sample ID number 447, paratype, MUB 63611	
PX606304	*idem*, 38.071584 N, 2.269359 W, 1782 m, 16 July 2025, *R.M. R.M. Ros, O. Werner &A. Calvo-Torralbo s.n.*, sample ID number 909, paratype, MUB 63615	
-	*idem*, 38.082104 N, 2.24175 W, 1540 m, 15 July 2025, *R.M. R.M. Ros, O. Werner &A. Calvo-Torralbo s.n.*, sample ID number 900, paratype, MUB 63612	
-	*idem*, 38.0722770 N, 2.2699746 W, 1759 m, 16 July 2025, *R.M. R.M. Ros, O. Werner &A. Calvo-Torralbo s.n.*, sample ID number 907, paratype, MUB 63613	
-	*idem*, 38.0722770 N, 2.2699746 W, 1759 m, 16 July 2025, *R.M. R.M. Ros, O. Werner &A. Calvo-Torralbo s.n.*, sample ID number 908), paratype, MUB 63614	
	idem, 38.070131 N, 2.264594, 1950 m, 16 July 2025, *R.M. R.M. Ros, O. Werner &A. Calvo-Torralbo s.n.*, sample ID number 912, paratype, MUB 63616	
	Tortula subulata* var.*graeffii	
PX606302	ITALY: Trentino-Alto Adige/Südtirol, Castelrotto/Kastelruth village, Hotel Belbedere-Schönblick wall, 46.565134 N, 11.556871 E, 3 August 2025, *R.M. Ros & O. Werner s.n.*, sample ID number 955, MUB 65501	√
AY934555	[Cano et al. 2005] MUB 17232	√
AY934556	[Cano et al. 2005] MUB 17230	√
AY934557	[Cano et al. 2005] MUB 14044	√
-	SWITZERLAND: Graubünden, Ober-Engadin, bei Pontresina, Juli 1883, *H. Graef s.n.*, lectotype, JE04007844	
-	ITALY: Trentino-Alto-Adige, Südtirol, Schnalstal/Val Senales, Katharinaberg, 46.689642 N, 10.935743 E, 1258 m, 29 July 2025, *R.M. R.M. Ros & O. Werner s.n.*, sample ID number 921, MUB 65486	
-	*idem*, sample ID number 923, MUB 65488	√
-	*idem*, sample ID number 924, MUB 65489	
-	*idem*, sample ID number 925, MUB 65490	
-	*idem*, sample ID number 926, MUB 65491	√
-	*idem*, sample ID number 927, MUB 65492	√
-	ITALY: Trentino-Alto Adige/Südtirol, Siusi allo Sciliar/Seis am Schlern, funicular station wall, 46.540039 N, 11.562976 E, 1003 m, 3 August 2025, *R.M. Ros & O. Werner s.n.*, sample ID number 949, MUB 65497	
-	*idem*, sample ID number 950, MUB 65498	
-	ITALY: Trentino-Alto Adige/Südtirol, Siusi allo Sciliar/Seis am Schlern, near hotel Salegg, 46.537448N, 11.557130 E 65499, 1037 m, 3 August	

	2025, *R.M. R.M. Ros & O. Werner s.n.*, sample ID number 952, MUB 65499	
-	ITALY: Trentino-Alto Adige/Südtirol, Castelrotto/Kastelruth village, Hotel Belbedere-Schönblick external wall, 46.565134 N, 11.556871 E, 3 August 2025, *R.M. R.M. Ros & O. Werner s.n.*, sample ID number 954, MUB 65500	√
-	ITALY: Trentino-Alto Adige/Südtirol, Castelrotto/Kastelruth village, small wall on the roundabout at the entrance to the village, junction of roads LS25 and LS24.46.556573 N, 11.556315 E, 3 August 2025, *R.M. Ros & O. Werner s.n.*, sample ID number 958, MUB 65503	
-	*idem*, sample ID number 959, MUB 65504	
-	*idem*, sample ID number 960, MUB 65505	
-	*idem*, sample ID number 961, MUB 65506	
-	NORWAY: Møre og Romsdal, Stranda, I skogen nordaust for ferjekaia i Geiranger, UTM32 V 406336,6887142, 100 m, 1 May 2015, *Kristin Wangen KW15072 & Torbjørn Høitomt*, TRH B-92986	
	Tortula subulata s.s.	
PX496597	SPAIN: Cuenca province, Serranía de Cuenca, road CM-2106, km 54, 40.393701 N, 1.892926 W, 1489 m, 28 Juni 2023, *R.M. Ros & O. Werner s.n.*, sample ID number 423, MUB 65509	
PX496598	GERMANY: Bayern, Haselbrunn, road BT 26, km 0,020, near Pottenstein, 49.787572 N, 11.422792 W, 430 m, 20 August 2024, *R.M. Ros & O. Werner s.n.*, sample ID number 556, MUB 65512	
PX496599	GERMANY: Bayern, Pottenstein, Mariental, parking area on the outskirts of the village, in the direction of Hasebrunn, 49.777340 N, 11.416693, 408 m, 20 August 2024, *R.M. Ros & O. Werner s.n.*, sample ID number 552, MUB 65511	
PX496600	SPAIN: Cuenca province, Serranía de Cuenca, road CM-2106, km 54, 40.393701 N, 1.892926 W, 1489 m, 28 Juni 2023, *R.M. Ros & O. Werner s.n.*, sample ID number 426, MUB 65510	
PX606303	SPAIN: Cuenca province, Serranía de Cuenca, road CM-2106, km 54, 40.393701 N, 1.892926 W, 1489 m, 28 Juni 2023, *R.M. Ros & O. Werner s.n.*, sample ID number 422, MUB 65508	
	SPAIN: Almería province, Sierra de Filabres, Calar Alto, near astronomical observatory, 37.220434 N, 2.536144 W, 2116 m, 9 April 2025, *R.M. Ros, O. Werner & A. Calvo-Torralbo s.n.*, sample ID number 764, MUB 65516	
